# Applying Cognitive Load Theory to Improve Instructional Efficiency and Learner Experience in Current Good Manufacturing Practices Training for Pharmacists

**DOI:** 10.3390/pharmacy14040105

**Published:** 2026-07-09

**Authors:** Russell D. Wilson, Paroma Arefin, Sujit S. Sansgiry

**Affiliations:** Department of Pharmaceutical Health Outcomes and Policy, College of Pharmacy, University of Houston, Houston, TX 77204, USA; rwilson6@uh.edu (R.D.W.); parefin@cougarnet.uh.edu (P.A.)

**Keywords:** cognitive load theory, Cognitive Theory of Multimedia Learning, current Good Manufacturing Practices (cGMP), instructional efficiency, learner experience, pharmacy education, instructional design, crossover study

## Abstract

Current Good Manufacturing Practice (cGMP) training is federally mandated for pharmaceutical manufacturing personnel and plays a critical role in ensuring product quality and patient safety. Despite extensive evidence supporting Cognitive Load Theory (CLT) and Mayer’s Cognitive Theory of Multimedia Learning (CTML), their application to cGMP training has not been evaluated. This study examined whether a CLT/CTML-informed cGMP video training strategy could improve instructional efficiency and learner experience among pharmacists. A randomized repeated-measures crossover study was conducted among 49 pharmacists. Participants completed both an enhanced CLT/CTML-informed training strategy and a standard training strategy in randomized order. Instructional efficiency, calculated from learner performance and perceived mental effort, and learner experience outcomes were assessed using paired-samples t-tests. Compared with the standard training strategy, the CLT/CTML-enhanced strategy resulted in higher assessment scores (78% vs. 72%, *p* = 0.04), lower perceived mental effort (2.4 vs. 3.5, *p* < 0.001), and greater instructional efficiency (0.50 vs. −0.50, *p* < 0.001). Learner experience ratings, including satisfaction, perceived knowledge gain, and likelihood of future recommendation, also improved significantly (all *p* < 0.001). Applying CLT and CTML principles to cGMP video training significantly improved instructional efficiency and learner experience, supporting theory-informed instructional design as a practical approach for complex regulatory education.

## 1. Introduction

Training in current Good Manufacturing Practices (cGMP) is mandated by federal law at all pharmaceutical manufacturing facilities, and pharmacists—who practice at the end of the pharmaceutical supply chain and routinely engage in compounding—are directly bound by these standards. the consequences of inadequate compliance are substantial [1]. A recent ten-year analysis of FDA data identified 3718 drug recall events over 2012–2023, with an increasing annual trend and cGMP control failures as a primary driver [2]. Another study confirmed that inadequate personnel training is a consistent root cause of these violations, establishing a direct link between training quality and patient safety [3]. Despite these stakes, cGMP training remains difficult to deliver effectively, including within pharmacy education and pharmacist professional development. The regulatory content is dense and is widely perceived by learners as abstract or tedious, precisely the conditions under which extraneous cognitive load impairs learning [4]. Compounding this, those selected to deliver cGMP training are typically chosen for regulatory expertise rather than instructional design skill, a gap noted in the literature for over three decades but largely unaddressed [5]. Consequently, improving the design and delivery of cGMP training for pharmacists and pharmacy learners is not merely an educational concern but a patient safety priority.

Cognitive Load Theory (CLT) offers a direct, evidence-based solution to this problem [6]. First articulated by Sweller in 1988, CLT holds that effective instructional design must account for the limited capacity of working memory [7,8]. CLT provides a framework for designing instruction by simultaneously considering the structure of the information being presented and the cognitive architecture through which learners process that information, thereby enabling the development of instructional strategies that facilitate learning while reducing unnecessary cognitive burden [9]. According to CLT, learning is most efficient when instructional materials minimize unnecessary cognitive burden, referred to as extraneous load, while directing cognitive resources toward meaningful knowledge construction (germane load) [9]. For example, instructional materials that require learners to search for relevant information or process unnecessary content may impose extraneous cognitive load, diverting working memory resources away from schema acquisition and meaningful learning [9]. Over four decades of empirical research have consistently demonstrated that CLT-informed instructional strategies improve both learner performance and satisfaction across diverse educational and the theory has been applied with notable success in health professions education settings [10,11,12]. Previous research in health professions education has demonstrated that instructional strategies informed by CLT and CTML can enhance knowledge retention, comprehension, and learner engagement while reducing cognitive load across a variety of educational formats, including asynchronous video and lecture-based instruction [13,14]. Despite growing recognition of the value of CLT within the pharmacy education literature, and repeated calls for evidence-based approaches to address pharmacy curriculum overload, CLT-informed instructional design has received relatively little empirical attention in pharmacy—and almost none in pharmacist regulatory training [15,16].

Because the present intervention was delivered via asynchronous video, a second, complementary framework is directly relevant: Mayer’s Cognitive Theory of Multimedia Learning (CTML), which extends CLT specifically to multimedia and video-based instruction [17]. CTML holds that learners process information through separate auditory and visual channels, each capacity-limited, and specifies 15 evidence-based design principles, including the coherence, signaling, redundancy, and modality principles for reducing extraneous processing [18]. In the present study, these principles were applied by reducing unnecessary on-screen information, emphasizing key regulatory concepts through visual cues, and integrating narration with relevant visuals to facilitate learning while minimizing unnecessary cognitive processing [18,19]. Together, CLT and CTML provide the theoretical foundation for the training strategy evaluated in this study. These complementary frameworks were selected because they provide evidence-based guidance for designing instructional videos that improve learning efficiency while reducing unnecessary cognitive burden, making them particularly well suited for complex regulatory topics such as cGMP. Because these frameworks aim to improve learning while reducing unnecessary cognitive burden, their effectiveness is appropriately assessed using measures that capture both learning outcomes and cognitive effort. Accordingly, we evaluated the intervention using two complementary outcomes. Instructional efficiency, the primary outcome, is a well-validated composite measure that integrates learner performance and perceived mental effort [20]. Instructional efficiency reflects how effectively learners perform relative to the mental effort required to achieve that performance, with higher instructional efficiency indicating better learning achieved with lower cognitive effort [20,21]. It has been used extensively in CLT research and captures both cognitive and performance dimensions of learning in a single interpretable index [20,21]. Together, instructional efficiency and learner experience provide complementary measures of educational effectiveness by capturing both objective learning outcomes and learners’ perceptions of the training intervention. In addition to objective learning outcomes, learner experience—comprising evaluation, overall satisfaction, perceived knowledge gain, and likelihood of recommendation- provides a complementary measure of subjective training quality, grounded in Kirkpatrick’s evaluation framework [22]. As pharmacist and health professional satisfaction with asynchronous e-learning is itself multidimensional, measuring these distinct dimensions is well justified [23]. Prior work confirms that CLT-informed redesign improves not only performance but also engagement and motivation, reinforcing the relevance of both outcomes in this study [14,18].

Although the use of generative AI in pharmacy education and practice is increasing [24], its application to regulatory compliance training remains limited. AI-generated content may contain inaccuracies, including incorrect regulatory citations, and does not inherently incorporate evidence-based cognitive load management principles such as CLT and CTML [11,25,26]. Because regulatory training requires accurate, verifiable, and audit-ready content [24], theory-informed instructional design remains essential. Although instructional design has been explored in cGMP training, the application of CLT and CTML to cGMP training has not been reported in published literature. Therefore, this study aimed to (1) develop an enhanced cGMP training strategy grounded in CLT and CTML principles for pharmacists and (2) evaluate its effects on instructional efficiency and learner experience using a randomized crossover design.

## 2. Materials and Methods

### 2.1. Study Design

A prospective, randomized, repeated-measures crossover design was employed. Participants were randomized to receive both training conditions (enhanced and control) in a counterbalanced sequence, with demographic information collected between exposures. By allowing each participant to serve as their own control, this within-subject design minimized the influence of interindividual variability in prior knowledge, learning preferences, and baseline familiarity with cGMP concepts. Consequently, the design enhanced statistical power while reducing the sample size required to detect meaningful differences between training strategies.

**Figure 1:**
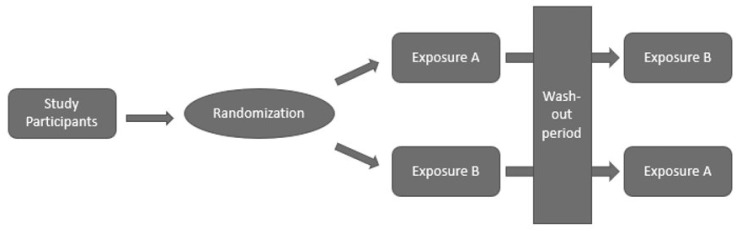
Randomized repeated measures crossover design used in the study.

Pharmacists were selected as the study population because of their critical role in ensuring medication quality and safety within the pharmaceutical supply chain, and their frequent involvement in compounding activities, which are subject to cGMP regulations. A schematic representation of the study design is provided in Figure 1.

### 2.2. Intervention Development

An enhanced cGMP training strategy was developed by systematically applying principles from CLT and CTML. The training was delivered as an asynchronous video presentation. Prior research has shown that enhanced asynchronous video design improves knowledge acquisition and recommendation rates in health professions settings [27]. Therefore, additional presentation design strategies were adopted from Reynolds’ Presentation Zen Design (2nd ed.) [28]. The enhanced training incorporated modifications to typography, imagery, text density, slide layout, signaling, and audio-visual integration, all intended to optimize learner engagement and reduce extraneous cognitive load [29]. A summary of these instructional design enhancements is provided in Table 1.

Content was drawn from two cGMP regulatory subparts: Subpart B—Organization and Personnel and Subpart F—Production and Process Control. These were selected for their regulatory importance and for being among the most frequently cited by the FDA in cGMP inspection violations. Both subparts were produced in both enhanced and control versions; each participant was randomized to receive one subpart in the enhanced format and the other in the control format, ensuring full within-subject exposure to both conditions.

The control training strategy consisted of videos presenting a textual display and voice narration of the subpart contents exactly as written in the federal regulations, representative of training commonly delivered in pharmaceutical industry settings or lecture-based pharmacy courses. Video length, key points covered, and introductory and concluding slides were kept identical across enhanced and control versions. Final videos are accessible at: https://youtu.be/owI6UvgUWl4 (accessed on 7 April 2026).

### 2.3. Outcome Measures

The primary outcome was instructional efficiency, a validated composite measure incorporating both learner performance and perceived mental effort [20]. Performance was assessed via a six-item True/False quiz covering material from the training videos, developed and validated through expert review and three iterative pilot studies. Perceived mental effort was assessed using a single item adapted from Paas et al., also validated during expert review [20]. Instructional efficiency was calculated by converting performance and mental effort scores to standardized z-scores and applying the Paas–Van Merriënboer formula: E = (ZPtest − ZEtest)/√2, where E represents instructional efficiency, ZPtest the standardized performance score, and ZEtest the standardized mental effort score [30]. Positive efficiency values indicate higher performance achieved with relatively lower mental effort, whereas negative values indicate lower performance relative to the effort invested.

The secondary outcome was learner experience, assessed using four scales adapted from Kirkpatrick’s training evaluation framework [20]: (1) Evaluation—five items assessing clarity, interest, thought provocation, attention, and visual appeal of the training; (2) Overall Satisfaction—a single item assessing general satisfaction; (3) Perceived Knowledge—a single item assessing perceived knowledge enhancement; and (4) Future Recommendation—a single item assessing willingness to recommend the training to others. All items used a five-point Likert scale (1 = Strongly Disagree to 5 = Strongly Agree). Participants completed all measures immediately following each video, before viewing the second training condition.

### 2.4. Instrument Validation and Sample Size

All questionnaire items and experimental procedures were validated through two rounds of expert feedback and three iterative pilot studies. Revisions based on pilot feedback included increasing item difficulty, standardizing all scale anchors to a uniform five-point format, and clarifying Qualtrics navigation instructions. Pilot data were also used to calculate the required sample size with G*Power version 3.1, targeting an effect size of 0.5 at α = 0.05, with adequate power for a paired-samples t-test. Based on pilot performance means (control: 72.3 ± 10; enhanced: 77.8 ± 10), an adequate sample size was estimated at approximately 45 participants.

### 2.5. Recruitment, Ethics, and Procedure

Ethical approval was obtained under exempt status from the Institutional Review Board at the institute. The study was conducted in accordance with the Declaration of Helsinki. Pharmacists were recruited via emails sent to publicly available pharmacy addresses in the Houston metropolitan area, supplemented by snowball sampling and in-person pharmacy visits. Informed consent was obtained from all participants prior to study enrollment. The cGMP training module was developed specifically for this research study and was not part of a routinely offered continuing education (CE) program. Participation was voluntary, and participants did not receive CE credit for completing the training.

Data collection commenced in January 2023. All study procedures were conducted online via Qualtrics (Qualtrics International Inc., Provo, UT, USA). After reviewing the consent document, participants viewed both training videos in randomized order and completed all outcome measures immediately following each video. Demographic data (age, sex, race, and device type) were collected between the two video exposures.

### 2.6. Statistical Analysis

Data was exported from Qualtrics and analyzed using IBM SPSS Statistics (Version 28; IBM Corp., Armonk, NY, USA). Prior to analysis, all observations were reviewed for completeness and accuracy; missing or anomalous data were excluded. Internal consistency of the five-item Evaluation scale was assessed using Cronbach’s alpha. Successful manipulation of cognitive load was verified by comparing mental effort scores across conditions. Paired samples t-tests were used to assess within-subject differences for all outcome variables, with statistical significance set at *p* < 0.05. Descriptive statistics (means and standard deviations) are reported for all variables.

## 3. Results

### 3.1. Sample Characteristics

A total of 49 pharmacists completed the study. Sample characteristics are presented in Table 2. The sample of pharmacists was predominantly female (73%) and primarily identified as White or Asian (80% combined). Most participants (80%) completed the survey on a computer or laptop. Mean age was 35 years (SD = 9.5). Participants rated their prior cGMP knowledge at 2.6/10 (SD = 2.1) and interest in the topic at 5.3/10 (SD = 1.9). Reliability analysis of the five-item Evaluation scale yielded a Cronbach’s alpha of 0.92, indicating high internal consistency. Successful manipulation of cognitive load was confirmed: perceived mental effort among pharmacists was significantly lower following the enhanced strategy compared with the control (2.4 ± 0.9 vs. 3.5 ± 0.9, *p* < 0.001).

### 3.2. Instructional Efficiency

Results for instructional efficiency variables are presented in Table 3. Among pharmacists, performance scores were significantly higher following the enhanced strategy (78 ± 17% vs. 72 ± 16%, *p* = 0.04), and mental effort scores were significantly lower (2.4 ± 0.9 vs. 3.5 ± 0.9, *p* < 0.001). The resulting instructional efficiency score was significantly higher for the enhanced strategy (0.50 vs. −0.50, *p* < 0.001), indicating better learning outcomes achieved by pharmacists with less cognitive effort.

### 3.3. Learner Experience

Learner experience results are presented in Table 4. Among pharmacists, statistically significant improvements were observed for all five evaluation items (*p* < 0.001): clarity of information (4.2 vs. 3.2), interest (4.1 vs. 2.5), thought provocation (3.9 vs. 2.4), attention (4.0 vs. 2.4), and visual appeal (4.3 vs. 2.5). Significant improvements were also found for overall satisfaction (4.0 vs. 2.6, *p* < 0.001), perceived knowledge (4.1 vs. 3.3, *p* < 0.001), and future recommendation (4.1 vs. 2.6, *p* < 0.001).

## 4. Discussion

The findings of this study demonstrate that applying CLT and CTML principles to cGMP training materials can improve both instructional efficiency and learner experience among pharmacists. These results are consistent with previous studies showing that reducing extraneous cognitive load enhances learning performance, decreases mental effort, and improves learner satisfaction across various educational settings [10,31]. Similar benefits have been reported among emergency medicine residents and health professions students, where CLT- and CTML-based instructional designs improved knowledge retention, comprehension, and learner motivation [13,14]. The present study extends this evidence base into pharmacist regulatory training—an area of increasing importance for pharmacy practice and education yet largely absent from the pharmacy education literature. With an average of 330 FDA drug recalls occurring annually, many of which are associated with cGMP control failures and inadequate personnel training, improvements in cGMP training quality may have important implications for pharmaceutical quality and patient safety at the population level [2].

The findings of this study support the use of asynchronous video as an effective modality for cGMP training of pharmacists. A randomized controlled trial in health professions education demonstrated that enhanced asynchronous video design led to significantly greater knowledge retention and higher recommendation rates than standard video formats [27]. These findings are consistent with the results of the present study and suggest that instructional design plays an important role in optimizing the effectiveness of video-based learning. The improvement in instructional efficiency observed in this study was driven by both higher performance scores and lower perceived mental effort. Although the improvement in performance was modest but statistically significant (*p* = 0.04), participants reported a substantial reduction in cognitive effort (*p* < 0.001). This finding is consistent with CLT, which posits that learning is more efficient when extraneous cognitive load is minimized, enabling pharmacists to achieve comparable or better outcomes with less mental effort [9]. The relatively larger effect observed for mental effort than for performance may be explained by the brief nature of the intervention, which consisted of a single training video, and the limited sensitivity of the six-item true/false assessment. The instructional enhancements incorporated into the redesigned training were based on established CTML principles [32]. These strategies are intended to reduce unnecessary cognitive processing and direct learner attention toward essential information. Consistent with this approach, Mayer’s synthesis of more than 200 experimental comparisons demonstrated significant learning benefits when these principles were applied to multimedia instruction [19].

The enhanced training also resulted in substantial improvements in learner experience among the participating pharmacists. Pharmacists reported significantly higher ratings for visual appeal, interest, satisfaction, perceived knowledge gain, and likelihood of recommending the training. In particular, ratings for visual appeal (4.3 vs. 2.5) and interest (4.1 vs. 2.5) were markedly higher in the enhanced training condition. These findings are consistent with CTML predictions that well-designed multimedia instruction can increase engagement while reducing the perception of cognitive effort [32,33]. Such improvements may be particularly important in pharmacy regulatory and compliance training, where pharmacists and pharmacy learners may have limited intrinsic motivation for the topic yet are required to process complex, information-dense content.

These findings carry practical implications for pharmacy education and health professions training more broadly. Pharmacy faculty and preceptors designing training modules covering complex regulatory, clinical, or scientific content can apply CLT and CTML principles—such as reducing slide text, using supporting visuals, and signaling key information—to improve both learning outcomes and learner satisfaction. These design changes do not require specialized technology or substantial additional resources, making them feasible across a range of educational settings. This scalability is particularly important given that pharmacy educators are already managing curriculum overload and seeking evidence-based strategies to reduce learner cognitive burden without sacrificing content depth [16]. Importantly, while AI tools are increasingly discussed as potential solutions for educational content generation, they cannot replicate the deliberate cognitive load management that CLT/CTML-informed design provides, nor can they guarantee the regulatory accuracy and audit traceability required in cGMP compliance training [24,25]. Human-designed, theory-informed training of the kind demonstrated here therefore remains essential in this regulatory domain. The observation that CLT redesign can improve both performance and subjective experience simultaneously is practically significant: instructional design that feels better to learners is more likely to be completed, revisited, and recommended to peers [34]. Furthermore, Mauldin demonstrated that applying CLT to a complex pharmacy course improved student investment and generated positive learner feedback, corroborating the present findings in a closely related discipline [23]. Bland et al. similarly found that redesigning pharmacology slides using CTML principles improved both student achievement and situational interest, directly paralleling the performance and learner experience improvements observed in the present study [17]. The improved satisfaction and perceived knowledge gain observed in this study align with broader evidence that positive learner experiences are associated with greater long-term retention and intrinsic motivation for continued learning [17,34]. The multidimensional nature of the learner experience improvements observed—spanning clarity, interest, visual appeal, satisfaction, and recommendation—is consistent with validated models of asynchronous e-learning satisfaction, which recognize these as distinct but interrelated constructs [23].

The present study responds directly to calls in the pharmacy education literature for broader application of CLT and contributes to a growing evidence base for CLT in health and behavioral training design. This study demonstrates that the theory is applicable not only to clinical skill training but also to regulatory and compliance education—an important and underserved domain in health professions curricula. The approach taken here, grounding video redesign in both CLT and CTML, offers a model that can be replicated in other regulatory domains, including controlled substance training, infection control, and biosafety compliance education.

This study has several strengths. The randomized repeated measures crossover design controls for individual differences in prior knowledge and experience, maximizing statistical power. The use of a validated composite outcome measure (instructional efficiency) captures both performance and perceived cognitive effort simultaneously. Instrument validation across two rounds of expert review and three pilot studies ensures content validity of the questionnaire items.

The study has some limitations. The study sample was recruited from the Houston metropolitan area through voluntary participation and may not fully represent the workforce that typically receives cGMP training in manufacturing settings, Although the sample shared several characteristics with the contemporary U.S. pharmacist workforce, including the predominance of female pharmacists, participants were younger (mean age 35 years compared with 41.8 years nationally) and included a higher proportion of Asian pharmacists than reported in a recent national sample of U.S. pharmacists [35]. Therefore, the generalizability of these findings to pharmacists practicing in other geographic regions or pharmaceutical manufacturing settings may be limited. Additionally, although CLT and CTML provide robust frameworks for optimizing instructional design, they primarily emphasize cognitive processes and do not explicitly account for non-cognitive influences on learning, such as learner motivation, emotional state, self-efficacy, or engagement [6]. Future studies integrating cognitive and motivational learning theories may provide a more comprehensive understanding of educational effectiveness. The crossover design, while a strength in controlling for individual differences, introduces the possibility of carryover effects; future studies should formally test for period and sequence effects and incorporate explicit washout periods between conditions. The use of two distinct regulatory subparts as content for the two conditions introduces a potential topic effect that cannot be fully disentangled from the training strategy effect, and replication with matched content across conditions is warranted. Finally, the study measured immediate retention; longitudinal follow-up is needed to determine whether observed benefits persist over time.

## 5. Conclusions

The application of CLT and CTML to cGMP training materials demonstrated significant improvements in instructional efficiency and learner experience among the sample of pharmacists. The enhanced training strategy produced better learning outcomes with lower cognitive effort and was rated more favorably across all dimensions of learner experience. These findings extend the evidence supporting theory-informed instructional design in health professions education, especially for pharmacists, and provide a practical, replicable approach for developing training on complex regulatory and compliance-related topics. Therefore, even modest improvements in training effectiveness achieved through accessible instructional design modifications may contribute to meaningful gains in pharmaceutical quality and patient safety within pharmacy practice. Future research should evaluate effects of individual CLT and CTML design principles, test the approach in larger and more diverse pharmacist and pharmacy populations, incorporate formal carryover analyses, and assess the long-term sustainability of the observed benefits. As AI continues to evolve, future studies should also investigate how AI-assisted content development can be integrated with evidence-based instructional design frameworks, such as CLT and CTML, to create more effective, scalable, and learner-centered approaches to pharmacy regulatory education.

## Figures and Tables

**Table 1:** Instructional design enhancements implemented in the enhanced training strategy.

Feature	Enhancement Applied
Font	96 pt (titles) and 36 pt (content) Rockwell typeface used throughout. Full-bleed photographs supporting the key content points of each slide (CTML ^1^: coherence and multimedia principles).
Images	
Video	Short clips in introduction and conclusion to establish relevance of content.
Audio	Voice narration throughout; background music in intro/outro (CTML: modality principle).
Text Reduction	On-screen text reduced to key points only; approximately 90% reduction vs. control (CTML: redundancy and coherence principles).
Use of Space	Slides decluttered; text occupying no more than one-third of slide area (CLT ^1^: extraneous load reduction).
Signaling	Key points emphasized with contrasting text color and underlining (CTML: signaling principle).

^1^ CTML = Cognitive Theory of Multimedia Learning; CLT = Cognitive Load Theory.

**Table 2:** Sample characteristics (*N* = 49).

Characteristic	Category	*n* (%)
Sex	Female	36 (73%)
	Male	13 (27%)
Race	White (Caucasian)	23 (47%)
	Asian	16 (33%)
	African American	3 (6%)
	Hispanic	3 (6%)
	Other	4 (8%)
Device	PC/Laptop	39 (80%)
	Smart Phone	10 (20%)
	Tablet/iPad	0 (0%)
Mean Age, years (SD) ^1^	—	35 (9.5)
Prior cGMP Knowledge (0–10 scale, SD) ^1^	—	2.6 (2.1)
Interest in cGMP (0–10 scale, SD)	—	5.3 (1.9)

^1^ cGMP = current Good Manufacturing Practices; SD = standard deviation.

**Table 3:** Instructional efficiency variables: mean scores by training strategy (*N* = 49).

Measure	Enhanced Strategy Mean (SD)	Control Strategy Mean (SD)	*p*-Value †
Mental Effort * (1–5 scale)	2.4 (0.9)	3.5 (0.9)	<0.001
Performance Score + (%)	78 (17)	72 (16)	0.04
Instructional Efficiency Score ×	0.50	−0.50	<0.001

* Rated on a 5-point scale (1 = Strongly Disagree to 5 = Strongly Agree); lower scores indicate lower perceived mental effort. + Percentage of correct answers on the assessment. × Calculated as E = (ZPtest − ZEtest)/√2; positive values indicate greater efficiency. † Paired-samples *t*-test.

**Table 4:** Learner experience variables: mean scores by training strategy (*N* = 49).

Measure	Enhanced Strategy Mean (SD)	Control Strategy Mean (SD)	*p*-Value †
Presented information clearly	4.2 (0.7)	3.2 (1.0)	<0.001
Was interesting	4.1 (0.8)	2.5 (0.9)	<0.001
Was thought provoking	3.9 (0.9)	2.4 (1.0)	<0.001
Kept attention throughout	4.0 (0.9)	2.4 (1.0)	<0.001
Was visually appealing	4.3 (0.7)	2.5 (1.0)	<0.001
Evaluation Total (5–25 scale) ^1^	20.4 (3.1)	13.0 (3.9)	<0.001
Overall Satisfaction ^2^	4.0 (0.8)	2.6 (1.0)	<0.001
Perceived Knowledge ^2^	4.1 (0.6)	3.3 (0.9)	<0.001
Future Recommendation ^2^	4.1 (0.8)	2.6 (1.0)	<0.001

^1^ Sum of the five evaluation items (Presented information clearly, Was interesting, Was thought provoking, Kept attention throughout, Was visually appealing); possible range 5–25. ^2^ Rated on a 5-point scale (1 = Strongly Disagree to 5 = Strongly Agree). † Paired-samples *t*-test.

## Data Availability

The data presented in this study are available on request from the corresponding author due to the data used in this study contain sensitive participant information and is subject to privacy and ethical restrictions. Therefore, the data is not publicly available.
